# Cisplatin-Induced Skeletal Muscle Atrophy: Biomolecular Mechanisms and the Protective Role of Exercise-Induced Myokines

**DOI:** 10.3390/biom15111495

**Published:** 2025-10-23

**Authors:** Miaomiao Xu, Xiaoguang Liu

**Affiliations:** 1 College of Physical Education, Guangdong University of Education, Guangzhou 510800, China; liuxg@gzsport.edu.cn; 2 College of Sports and Health, Guangzhou Sport University, Guangzhou 510500, China

**Keywords:** chemotherapy, skeletal muscle atrophy, cisplatin, exercise intervention, myokines

## Abstract

Cisplatin is a widely used chemotherapy drug for the treatment of various cancers; however, its clinical use is often accompanied by skeletal muscle atrophy, which not only impacts patients’ physical health but also significantly diminishes their quality of life. The mechanisms underlying cisplatin-induced muscle atrophy are complex and involve a series of molecular biological processes, including oxidative stress, inflammation, protein degradation, and muscle cell apoptosis. Recent studies have suggested that exercise intervention can significantly alleviate cisplatin-induced muscle damage by modulating exercise-induced myokines. Myokines, such as muscle-derived cytokines (e.g., IL-6, irisin) and other related factors, can mitigate muscle atrophy through anti-inflammatory, antioxidative, and muscle-synthesis-promoting mechanisms. This review explores the molecular mechanisms of cisplatin-induced skeletal muscle atrophy, examines the potential protective effects of exercise intervention, and highlights the role of exercise-induced myokines in this process. The findings suggest that exercise not only alleviates chemotherapy-induced muscle atrophy by improving metabolic and immune status but also activates myokines to promote muscle regeneration and repair, offering a promising adjunctive therapy for cisplatin-treated patients.

## 1. Introduction

Skeletal muscle, which accounts for nearly 40% of total body mass, is fundamental to locomotion, metabolic regulation, and systemic homeostasis [1,2]. The maintenance of skeletal muscle mass and function is therefore critical for health and survival [3,4]. Muscle atrophy, defined as the loss of muscle mass and strength, is a debilitating condition that occurs in diverse clinical contexts, including aging [5], immobilization [6], chronic diseases [7,8], and cancer therapy [9]. In oncology, muscle wasting has attracted particular concern because it worsens fatigue, reduces treatment tolerance, compromises immune competence, and is associated with poor prognosis [10,11,12].

Chemotherapy is a major contributor to secondary muscle atrophy [13,14,15]. Cisplatin, a platinum-based chemotherapeutic drug widely used against solid tumors [16], is especially notorious for inducing skeletal muscle loss [17,18]. Both preclinical and clinical studies demonstrate that cisplatin treatment rapidly accelerates muscle wasting [19,20,21,22,23]. Mechanistically, this process involves the overactivation of proteolytic systems such as the ubiquitin–proteasome [24] and autophagy–lysosome pathways [25], inhibition of protein synthesis due to dysregulated the protein kinase B (Akt)/mechanistic target of rapamycin (mTOR) signaling pathway [26,27], mitochondrial dysfunction with excessive reactive oxygen species (ROS) production [16,26], and chronic inflammatory activation [18]. Together, these alterations disrupt protein homeostasis and cellular metabolism, leading to progressive muscle degradation.

In this review, it is important to differentiate among cachexia, sarcopenia, and atrophy. Cachexia is a multifactorial metabolic syndrome associated with chronic diseases such as cancer, characterized by severe weight and muscle loss driven by systemic inflammation and metabolic imbalance [10]. Sarcopenia refers to the age-related, progressive decline in skeletal muscle mass and function, primarily due to reduced physical activity, hormonal alterations, and impaired protein synthesis [28]. In contrast, atrophy denotes localized muscle loss caused by disuse, denervation, or direct cytotoxic injury, such as that induced by chemotherapy [6,26]. Understanding these distinctions provides a conceptual framework for interpreting chemotherapy-induced muscle wasting within its unique pathological context.

Given the lack of effective pharmacological therapies [13,29], exercise has emerged as a promising non-pharmacological intervention to counteract chemotherapy-induced muscle wasting [30,31,32,33,34,35]. Different modes of exercise act through distinct biomolecular pathways. Endurance exercise enhances mitochondrial quality control and reduces systemic inflammation [36,37,38,39], whereas resistance training promotes muscle hypertrophy by stimulating protein synthesis and inhibiting proteolysis [40,41,42]. Importantly, exercise also induces the secretion of myokines—muscle-derived cytokines and peptides—that mediate local and systemic protective effects [43,44]. For example, irisin exerts anti-inflammatory and antioxidative actions that alleviate ROS-mediated damage [45,46,47]; interleukin-15 (IL-15) stimulates protein synthesis and suppresses catabolic signaling [48,49]; and brain-derived neurotrophic factor (BDNF) supports mitochondrial biogenesis and neuromuscular junction integrity [50,51]. Through these mechanisms, exercise-induced myokines provide a molecular link between physical activity and the preservation of muscle mass under chemotherapeutic stress.

The purpose of this review is to summarize the molecular mechanisms of chemotherapy-induced skeletal muscle atrophy, with a particular focus on cisplatin-related pathways, and to discuss how exercise counteracts these detrimental changes. We highlight the roles of different exercise modalities and their associated myokine responses, and we provide perspectives on future research and translational implications. Understanding these biomolecular interactions may guide the development of integrative therapeutic strategies aimed at preserving skeletal muscle health in patients undergoing chemotherapy.

## 2. Methods

This review aims to provide a comprehensive synthesis of recent advances in understanding cisplatin-induced skeletal muscle atrophy and the protective effects of exercise-based interventions. A systematic literature search was conducted to identify relevant studies published from January 2020 to September 2025 across PubMed, Web of Science, Scopus, and Google Scholar.

The search strategy combined controlled vocabulary (e.g., MeSH terms) and free-text keywords to identify relevant studies on chemotherapy-induced muscle wasting, cisplatin toxicity, and exercise interventions. The search focused on studies exploring the molecular mechanisms underlying cisplatin-induced skeletal muscle atrophy and the protective effects of exercise, particularly how exercise-induced myokines and related pathways contribute to muscle preservation. Key terms included “Cisplatin,” “Chemotherapy-induced muscle atrophy,” “Cachexia,” “Skeletal muscle wasting,” “Exercise,” “Endurance training,” “Resistance training,” “Combined exercise,” “Myokines,” “IL-6,” “Irisin,” “Mitochondrial dysfunction,” “Oxidative stress,” “Proteostasis,” “Inflammation,” “Muscle regeneration,” “Muscle synthesis,” “Apoptosis,” “Muscle injury repair,” “Cisplatin toxicity mechanism,” “Aerobic exercise,” and “Anaerobic exercise.” To provide a more complete molecular perspective on muscle atrophy and adaptation, additional signaling-related keywords such as Akt, mTOR, MuRF-1, LC3B, and PGC1α were also considered. Boolean operators (AND, OR) and truncation techniques were applied to optimize sensitivity and specificity.

Studies were included if they met the following criteria: (1) peer-reviewed publications in English; (2) human or animal studies, including randomized controlled trials, observational studies, cohort studies, and preclinical experimental studies; (3) primary focus on cisplatin-induced muscle atrophy, chemotherapy-related cachexia, or exercise-based protective mechanisms; and (4) relevance to molecular pathways such as protein turnover, mitochondrial biology, oxidative stress, or inflammation, as well as exercise modalities including endurance, resistance, or combined training. Exclusion criteria comprised non-English publications, preprints or grey literature, in vitro studies without in vivo validation, and articles lacking clear relevance to the review objectives.

Although the review was limited to English-language publications, it is acknowledged that relevant studies may also exist in other languages such as Chinese, Russian, French, or German. Many non-English publications include English abstracts, so this language restriction may have excluded some valuable evidence and should be considered a potential limitation of the study.

**Figure 1:**
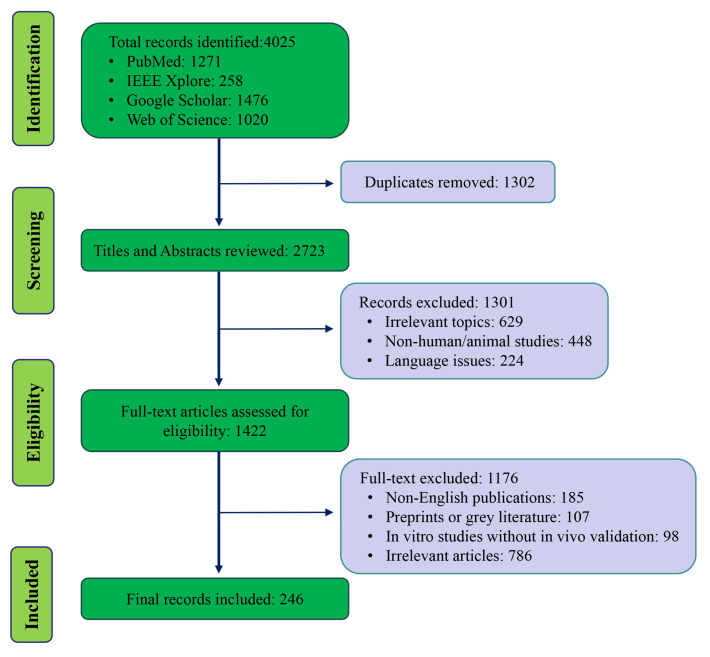
Literature selection flow diagram.

A total of 246 articles were identified and screened based on relevance and inclusion criteria. The screening process involved an initial review of titles and abstracts, followed by full-text screening to confirm eligibility. A PRISMA-like flow diagram outlining the study selection process is provided below to increase transparency (Figure 1).

## 3. Chemotherapy-Induced Skeletal Muscle Atrophy

### 3.1. Clinical Relevance of Chemotherapy-Associated Muscle Wasting

Cancer patients frequently experience muscle wasting during the course of treatment, which severely affects their functional capacity, quality of life, and overall survival [9,52]. Chemotherapy has been identified as one of the main contributors to secondary muscle atrophy, independent of tumor burden itself [53,54,55]. The phenomenon is often described as chemotherapy-induced cachexia or sarcopenia, characterized by decreased muscle mass, weakness, and metabolic dysfunction [56,57]. Clinical observations demonstrate that patients undergoing platinum-based chemotherapy, including cisplatin, exhibit accelerated loss of lean body mass compared with non-treated cancer patients [58,59].

This muscle wasting is clinically significant for several reasons. First, reduced muscle mass compromises treatment tolerance, leading to increased dose-limiting toxicities, treatment delays, or discontinuation [56,60,61]. Second, skeletal muscle depletion negatively impacts the pharmacokinetics of chemotherapeutic drugs, potentially altering efficacy and increasing systemic toxicity [62,63]. Third, muscle wasting worsens cancer-related fatigue, a major determinant of patient well-being [64,65,66]. Finally, low muscle mass is consistently associated with poor overall survival and disease-free survival in multiple cancer types [67,68,69,70,71,72]. These findings underscore the urgent need for strategies to mitigate chemotherapy-induced skeletal muscle atrophy in clinical settings.

### 3.2. Cisplatin and Skeletal Muscle Atrophy: Clinical and Preclinical Evidence

Among various chemotherapeutic agents, cisplatin has been extensively studied for its muscle-wasting effects [16,18]. Clinically, cisplatin-based regimens are associated with rapid loss of lean mass, even within the first few cycles of treatment [56,73]. Cross-sectional and longitudinal studies show that cancer patients treated with cisplatin present with reduced muscle cross-sectional area and decreased grip strength, both of which predict poor clinical outcomes [57,74].

Preclinical studies using rodent models have provided valuable insights into the phenotypic changes associated with cisplatin-induced muscle wasting [21,75,76]. Both Liu et al. and Seo et al. found that cisplatin administration leads to significant reductions in body weight and muscle mass, particularly in fast-twitch muscles such as the gastrocnemius and tibialis anterior [18,77]. Histological analysis reveals muscle fiber atrophy and an increase in the expression of atrophy-related genes, including Atrogin-1 (also known as FBXO32) and muscle RING finger-1 (MuRF1), both of which play key roles in muscle degradation [18,56,73]. Additionally, cisplatin induces mitochondrial dysfunction and oxidative stress [78]. In mice, cisplatin impairs mitochondrial respiratory capacity, decreases adenosine triphosphate (ATP) production, and elevates ROS levels, which contribute to oxidative damage in muscle fibers [18,77,79].

In addition to these well-established catabolic pathways, recent studies have highlighted the pivotal role of glycogen synthase kinase 3 beta (GSK3β) in cisplatin-induced muscle atrophy [16]. GSK3β, which can be activated by cisplatin treatment, contributes to skeletal muscle loss by suppressing ribosomal RNA synthesis, impairing mitochondrial function, and promoting muscle fiber degradation [16,77,80,81]. Moreover, GSK3β acts as a key regulator in the Wingless/Integrated–β-catenin signaling cascade, where its overactivation accelerates β-catenin degradation, thereby inhibiting myogenic differentiation and protein synthesis [82,83]. Exercise interventions and GSK3β-targeted therapies have been reported to attenuate GSK3β activity and restore β-catenin signaling, improving muscle regeneration and function [84,85].

Although the molecular mechanisms underlying muscle atrophy share several common pathways across different etiologies, cisplatin-induced skeletal muscle wasting exhibits unique features that distinguish it from other forms such as disuse, denervation, or cancer cachexia [86]. Specifically, cisplatin directly induces mitochondrial DNA damage and oxidative stress through cisplatin–DNA adduct formation [17], which rarely occurs in other atrophy models [18]. In addition, chemotherapy-related systemic inflammation (elevated IL-6 and TNF-α) [77,87] and suppressed anabolic myokine secretion (e.g., IL-15, irisin) [88] collectively exacerbate protein degradation [20]. Moreover, cisplatin markedly inhibits proliferator-activated receptor gamma coactivator 1-alpha (PGC-1α) expression and mitochondrial biogenesis [16], leading to energy metabolism failure and impaired muscle regeneration [75]. These features reflect a dual mechanism involving both direct cytotoxic injury to myofibers and systemic metabolic dysregulation, highlighting that cisplatin-induced atrophy represents a distinct biological entity, with its major comparative characteristics summarized in Table 1 [89].

Taken together, clinical and preclinical evidence strongly supports the role of cisplatin in promoting skeletal muscle atrophy through multifactorial biomolecular mechanisms [20,92,93,94] (Table 2). Understanding these mechanisms provides a foundation for exploring targeted interventions, such as exercise, to counteract cisplatin-induced muscle wasting [16,95].

### 3.3. Chemotherapy-Induced Decreased Motility and Secondary Muscle Disturbance

Beyond the direct cytotoxic effects of cisplatin on skeletal muscle, chemotherapy frequently reduces patients’ physical activity due to fatigue, nausea, and overall malaise [64]. This treatment-induced decline in mobility promotes a secondary form of disuse atrophy, which synergistically exacerbates cisplatin-induced muscle loss [96]. Reduced mechanical loading further impairs mitochondrial turnover and oxidative capacity [97], suppresses muscle protein synthesis [98], and activates proteolytic systems such as the ubiquitin–proteasome and autophagy–lysosome pathways [41]. In addition, inactivity-driven metabolic inflexibility amplifies the pre-existing energy deficit caused by cisplatin-mediated mitochondrial dysfunction [79], resulting in accelerated muscle catabolism [20]. Collectively, the combined effects of chemotherapy-related inactivity and cisplatin toxicity create a feed-forward cycle of muscular and metabolic deterioration [35]. These insights emphasize the importance of incorporating early-stage physical rehabilitation or mild exercise interventions to mitigate both primary and secondary muscle atrophy during chemotherapy [99].

## 4. Molecular Mechanisms Underlying Muscle Atrophy

**Figure 2:**
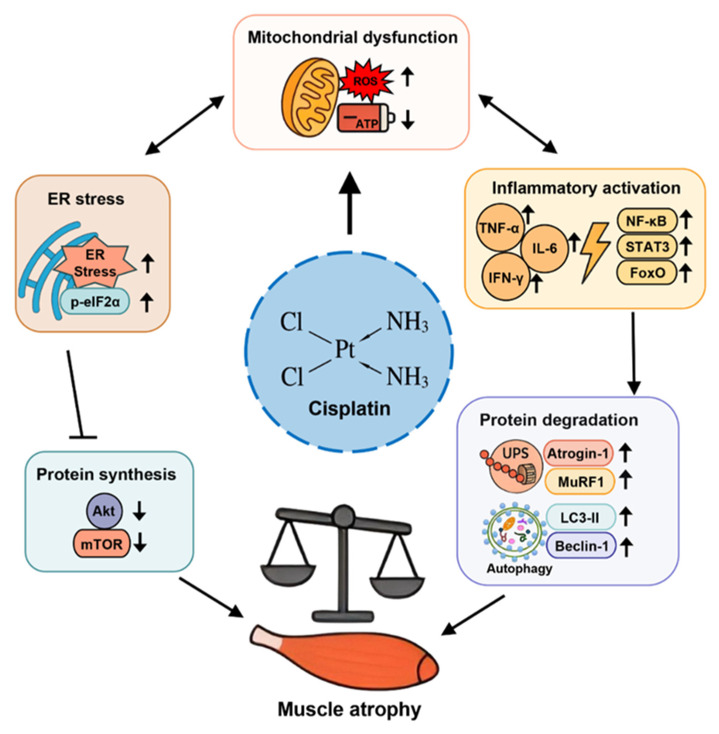
Overview of molecular mechanisms underlying cisplatin-induced muscle atrophy. Cisplatin induces mitochondrial dysfunction, endoplasmic reticulum stress, and inflammatory responses, which activate the ubiquitin–proteasome system and autophagy, thereby enhancing protein degradation. At the same time, cisplatin suppresses the Akt/mTOR signaling pathway, leading to reduced protein synthesis. The imbalance between protein degradation and synthesis ultimately results in muscle atrophy. In the figure, “↑” denotes upregulation or activation, “↓” indicates downregulation or suppression, and “⊥” represents inhibitory regulation. ROS, reactive oxygen species; ER, endoplasmic reticulum; UPS, ubiquitin–proteasome system; LC3-II, microtubule-associated protein 1 light chain 3-II; mTOR, mechanistic target of rapamycin; Akt, protein kinase B; NF-κB, nuclear factor kappa B; STAT3, signal transducer and activator of transcription 3; FoxO, forkhead box O; TNF-α, tumor necrosis factor alpha; IL-6, interleukin-6; IFN-γ, interferon gamma.

Skeletal muscle mass is determined by the dynamic balance between protein synthesis and degradation [100,101]. Chemotherapy-induced muscle wasting, particularly that caused by cisplatin, results from a disruption of this balance through multiple converging molecular pathways [17]. The major mechanisms include activation of proteolytic systems, suppression of anabolic signaling, mitochondrial dysfunction, oxidative stress, and inflammatory dysregulation [98,102]. Figure 2 illustrates these key molecular mechanisms and how they interact to contribute to cisplatin-induced muscle atrophy.

### 4.1. Protein Degradation Pathways

One of the hallmarks of cisplatin-induced muscle atrophy is the activation of protein degradation systems [103,104]. The ubiquitin–proteasome system (UPS) plays a central role in the breakdown of myofibrillar proteins [16]. Two muscle-specific E3 ubiquitin ligases, Atrogin-1 and MuRF1, are consistently upregulated in cisplatin-treated muscle [105,106]. Their activation enhances ubiquitination and subsequent proteasomal degradation of structural proteins such as myosin heavy chain, leading to muscle fiber shrinkage [107,108].

In parallel, the autophagy–lysosome system is also stimulated [109]. Cisplatin increases autophagy-related proteins, including microtubule-associated protein 1A/1B-light chain 3-II (LC3-II) and Beclin-1, promoting excessive autophagic flux [77,110]. While basal autophagy is essential for cellular homeostasis, sustained overactivation contributes to muscle loss by degrading organelles and cytoplasmic components [111,112]. Together, these pathways shift protein turnover toward catabolism, thereby driving muscle wasting.

### 4.2. Suppression of Protein Synthesis

In addition to enhanced degradation, cisplatin suppresses protein synthesis by inhibiting the Akt/mTOR signaling pathway [17,29,105,113,114]. Under normal conditions, Akt activation stimulates mammalian target of rapamycin complex 1 (mTORC1), which in turn promotes ribosomal biogenesis and translation initiation [115,116]. However, cisplatin reduces Akt phosphorylation and mTOR activity, leading to decreased expression of downstream effectors such as p70S6 kinase and eukaryotic translation initiation factor 4E-binding protein 1 (4E-BP1) [17,117]. This impairment of anabolic signaling reduces the ability of muscle fibers to maintain protein mass.

Furthermore, cisplatin induces endoplasmic reticulum (ER) stress, which activates eukaryotic translation initiation factor 2 subunit alpha (eIF2α) phosphorylation and suppresses global protein translation [118,119,120]. The combined effects of reduced mTOR signaling and ER stress-mediated translational arrest exacerbate the imbalance between synthesis and degradation [121].

### 4.3. Mitochondrial Dysfunction and Oxidative Stress

Mitochondrial impairment is another critical factor in cisplatin-induced muscle atrophy [76,86]. Cisplatin damages mitochondrial deoxyribonucleic acid (DNA), disrupts the electron transport chain, and decreases ATP production [77,122,123]. This energy deficit compromises muscle contractility and cellular metabolism [20]. At the same time, cisplatin increases the generation of ROS, which causes oxidative damage to lipids, proteins, and DNA [17,124,125].

Oxidative stress also activates catabolic signaling pathways [126]. For instance, ROS can stimulate the transcription factor forkhead box O (FoxO), which upregulates Atrogin-1 and MuRF1 [41,127]. In addition, mitochondrial dysfunction triggers mitophagy, contributing to excessive organelle degradation [97,128]. The vicious cycle of mitochondrial damage and ROS overproduction thus exacerbates muscle wasting [79].

### 4.4. Inflammatory Signaling

Cisplatin administration is associated with systemic and local inflammation [129]. Elevated levels of pro-inflammatory cytokines such as TNF-α, IL-6, and interferon-gamma (IFN-γ) are commonly observed [10,18,20,74]. These cytokines activate intracellular pathways including nuclear factor kappa-light-chain-enhancer of activated B cells (NF-κB) [130] and the janus kinase/signal transducer and activator of transcription 3 (JAK/STAT3) signaling pathway [131,132], which promote proteolysis and inhibit protein synthesis [41]. For example, NF-κB activation enhances the expression of Atrogin-1 and MuRF1 [1], while STAT3 activation contributes to mitochondrial dysfunction and metabolic reprogramming [133].

Inflammation also disrupts the function of regulatory immune cells such as Tregs, reducing their ability to maintain an anti-inflammatory environment [10,134]. The resulting imbalance between pro- and anti-inflammatory signaling accelerates muscle loss and contributes to systemic cachexia [133,135].

### 4.5. Crosstalk Between These Pathways

These molecular mechanisms do not operate in isolation but rather form a complex regulatory network [136,137]. For example, oxidative stress can activate NF-κB signaling, linking mitochondrial dysfunction to inflammation [138,139,140]. Similarly, inflammatory cytokines suppress Akt/mTOR signaling, thereby coupling inflammation with reduced protein synthesis [41,141]. ER stress also interacts with ROS generation and autophagic responses, further amplifying catabolic processes [142,143].

This extensive crosstalk suggests that chemotherapy-induced skeletal muscle atrophy is a multifactorial process, in which proteostasis imbalance, mitochondrial dysfunction, oxidative stress, and inflammation reinforce each other [35,97]. Targeting multiple nodes of this network, such as through exercise interventions, may therefore offer more effective protection against muscle wasting than single-pathway approaches [13,144].

## 5. Exercise as a Protective Intervention

Exercise has emerged as a promising non-pharmacological approach to counteract chemotherapy-induced skeletal muscle atrophy [13,145]. Unlike pharmacological interventions that typically target single molecules, exercise exerts multi-systemic effects that simultaneously influence protein turnover, mitochondrial function, oxidative stress, and inflammatory signaling [146,147]. Different exercise modalities, including endurance and resistance training, as well as their combination, confer distinct but complementary molecular benefits [148,149,150,151]. In addition, exercise induces the secretion of myokines, which mediate local and systemic protective effects [43,152,153].

### 5.1. Endurance Exercise and Mitochondrial Adaptations

Endurance exercise, such as treadmill running or swimming, enhances oxidative metabolism and mitochondrial quality control [154,155,156,157,158]. Preclinical studies show that endurance training improves mitochondrial biogenesis through activation of the PGC-1α pathway, thereby restoring ATP production and reducing cisplatin-induced mitochondrial dysfunction [19,105,159]. Enhanced mitochondrial turnover also decreases ROS accumulation, alleviating oxidative damage to muscle fibers [102,160,161].

**Figure 3:**
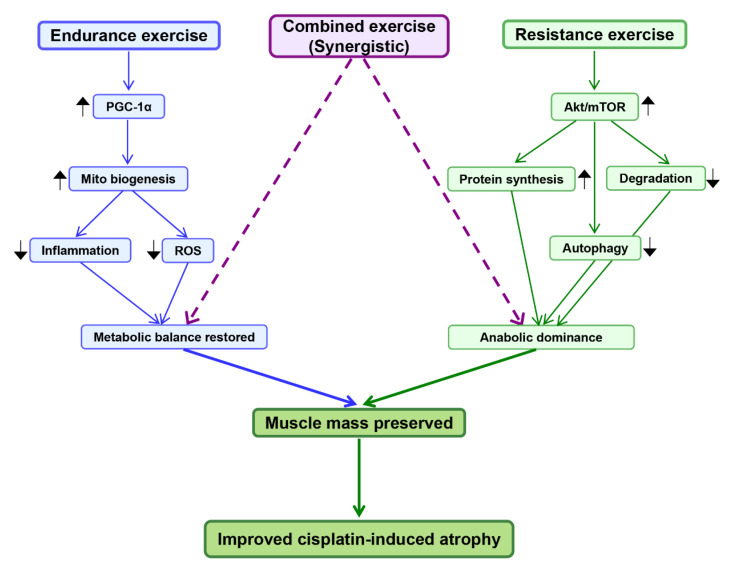
Protective effects of exercise intervention against cisplatin-induced muscle atrophy. Endurance exercise enhances mitochondrial biogenesis via PGC-1α, reduces ROS and inflammation, and restores metabolic balance. Resistance exercise activates Akt/mTOR signaling, promotes protein synthesis, inhibits degradation and excessive autophagy, leading to anabolic dominance. Combined exercise exerts synergistic benefits by integrating metabolic restoration and anabolic signaling. Together, these effects preserve muscle mass and attenuate cisplatin-induced atrophy. Arrows indicate regulation: ↑ upregulation/activation; ↓ downregulation/inhibition. Blue lines represent endurance pathways, green lines resistance pathways, and purple dashed lines synergistic interactions. PGC-1α, peroxisome proliferator-activated receptor gamma coactivator 1-alpha; ROS, reactive oxygen species; Akt, protein kinase B; mTOR, mechanistic target of rapamycin.

Endurance exercise further exerts systemic anti-inflammatory effects [162,163]. It downregulates pro-inflammatory cytokines such as TNF-α and IL-6, while promoting anti-inflammatory mediators including IL-10 [164,165]. These changes help to restore immune balance and attenuate the NF-κB and JAK/STAT3 pathways that drive catabolism [165,166]. Thus, endurance exercise addresses two major mechanisms of cisplatin-induced muscle wasting: mitochondrial dysfunction and chronic inflammation [77,105] (Figure 3).

### 5.2. Resistance Training and Anabolic Signaling

Resistance exercise is the most effective stimulus for promoting muscle hypertrophy and maintaining lean mass [167,168,169,170]. Mechanistically, resistance training activates the Akt/mTOR pathway, leading to increased phosphorylation of mTORC1 downstream effectors such as p70 ribosomal S6 kinase (p70S6K) and 4E-BP1 [42,171]. This activation enhances ribosomal biogenesis and translation initiation, counteracting cisplatin-induced suppression of protein synthesis [172,173].

Resistance training also inhibits proteolysis [174,175]. It reduces the expression of Atrogin-1 and MuRF1, thereby attenuating ubiquitin–proteasome-mediated degradation [102,176,177]. Additionally, resistance exercise has been shown to suppress excessive autophagy by normalizing LC3-II and Beclin-1 levels [178,179]. Together, these adaptations shift the balance toward protein anabolism, providing structural protection against muscle atrophy (Figure 3).

### 5.3. Combined Exercise and Synergistic Effects

While endurance and resistance training exert distinct benefits, their combination may produce synergistic effects [180,181,182]. Clinical studies in cancer survivors demonstrate that combined training improves both aerobic capacity and muscle strength [183,184]. In preclinical models, concurrent exercise has been shown to simultaneously enhance mitochondrial function, reduce ROS, activate mTOR signaling, and suppress catabolic gene expression [185,186].

Importantly, combined training may provide superior functional outcomes by integrating systemic metabolic improvements with structural muscle preservation [183,187] (Figure 3). This suggests that multimodal exercise prescriptions could be particularly beneficial for patients undergoing cisplatin-based chemotherapy [188,189].

### 5.4. Myokines as Mediators of Exercise-Induced Protection

**Figure 4:**
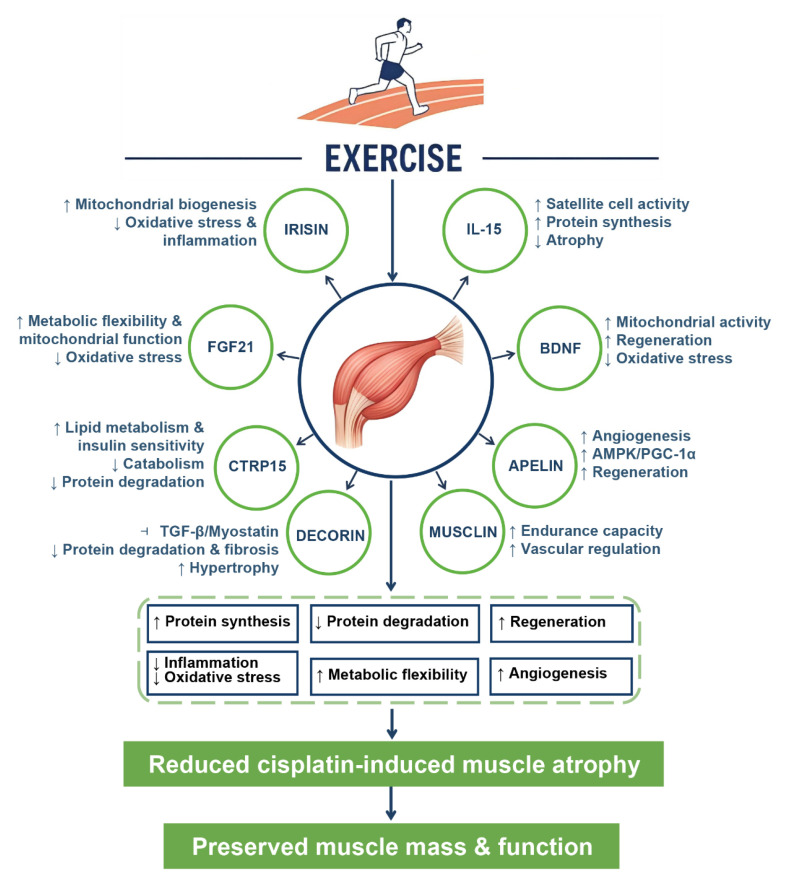
Role of exercise-induced myokines in counteracting cisplatin-induced muscle atrophy. The schematic illustrates how exercise promotes the release of multiple myokines from skeletal muscle, including Irisin, IL-15, BDNF, FGF21, Apelin, Decorin, CTRP15 (Myonectin), and Musclin. These myokines exert distinct biological effects by regulating mitochondrial biogenesis, protein turnover, metabolic flexibility, angiogenesis, and regenerative processes. Downstream, they converge on six protective mechanisms: inflammation and oxidative stress, protein synthesis, protein degradation, regeneration, angiogenesis, and metabolic flexibility, ultimately leading to reduced cisplatin-induced muscle atrophy and preserved muscle mass and function. ↑ indicate stimulation or increase; ↓ indicate reduction or suppression; ⊣ represent inhibition of signaling pathways. IL-15, interleukin-15; BDNF, brain-derived neurotrophic factor; FGF21, fibroblast growth factor 21; CTRP15, C1q/TNF-related protein 15 (Myonectin); AMPK, AMP-activated protein kinase; PGC-1α, peroxisome proliferator-activated receptor gamma coactivator-1α; TGF-β, transforming growth factor-beta.

Exercise protects skeletal muscle through the secretion of myokines, muscle-derived cytokines and peptides with endocrine-like actions [43,190,191]. These myokines circulate in the blood and not only support muscle cells but also influence other organs such as the liver, fat tissue, and brain [192,193]. Classic myokines, like irisin, IL-15, and BDNF, have been shown to reduce oxidative stress, stimulate protein synthesis, and promote mitochondrial biogenesis, forming a solid foundation for exercise-induced muscle protection [49,51,194]. Figure 4 shows how these myokines, such as irisin, IL-15, and BDNF, help reduce oxidative stress, promote protein synthesis, and improve mitochondrial function, providing protection against muscle atrophy. Irisin, in particular, is released during endurance exercise, enhancing mitochondrial function and energy production, which helps buffer energy deficits and prevent muscle degradation [46]. It also reduces oxidative stress and inflammation, offering protection against muscle wasting conditions, such as those induced by chemotherapy [45]. IL-15 is known to stimulate satellite cell activity, promote protein synthesis, and prevent muscle atrophy, while also reducing oxidative stress and inflammation [48]. BDNF increases after exercise and supports mitochondrial activity, protein synthesis, and muscle regeneration, helping to maintain muscle integrity under stress [195]. These classic myokines play a crucial role in muscle adaptation to exercise and offer potential therapeutic benefits for conditions like chemotherapy-induced muscle wasting.

Beyond these traditional factors, recent studies have identified novel myokines with potential relevance to chemotherapy-induced muscle wasting [89,196] (Figure 4). Fibroblast growth factor 21 (FGF21), a stress-inducible myokine elevated by exercise, enhances mitochondrial function and metabolic flexibility, thereby buffering the energy deficits associated with cisplatin treatment [197,198]. Apelin, whose secretion increases after endurance exercise, improves angiogenesis, activates AMPK/PGC-1α signaling, and stimulates satellite cell activity, facilitating muscle regeneration under wasting conditions [199,200,201]. Decorin, induced by resistance exercise, antagonizes transforming growth factor-beta (TGF-β) signaling while promoting hypertrophy, thus offering both antifibrotic and anabolic protection against chemotherapy-related muscle degeneration [202,203,204]. Emerging candidates such as myonectin (CTRP15) and musclin (osteocrin) have been implicated in lipid metabolism, vascular regulation, and endurance enhancement, though their precise roles in chemotherapy-induced muscle loss remain to be clarified [205,206].

Together, these classical and novel myokines highlight the ability of exercise to orchestrate a multi-system defense network, integrating redox control, proteostasis, angiogenesis, and regenerative pathways [191,207]. This perspective not only deepens mechanistic insights but also points to myokines as potential biomarkers and therapeutic targets to mimic the systemic benefits of exercise [201,207,208,209].

### 5.5. Summary of Protective Mechanisms

In summary, exercise mitigates chemotherapy-induced skeletal muscle atrophy through multiple converging mechanisms [210,211,212]. Endurance exercise improves mitochondrial function and reduces inflammation [196,213]; resistance training restores anabolic signaling and inhibits proteolysis [31,146]; combined training integrates these benefits to optimize outcomes [31,196]; and myokines serve as systemic mediators that amplify local adaptations [89,146]. These multifaceted effects highlight the unique potential of exercise as an integrative intervention to preserve skeletal muscle health in cancer patients.

## 6. Future Directions and Challenges

Although numerous studies have investigated the effects of cisplatin and exercise interventions using animal models [214,215], these findings may not fully translate to human physiology. Animal experiments allow for precise control of dosage, environment, and genetic background, but they cannot fully replicate the complexity of human responses, including interindividual variability, comorbidities, and lifestyle factors [214,216]. For example, several protective mechanisms of exercise observed in rodents—such as enhanced mitochondrial biogenesis and antioxidative enzyme activity—may differ in magnitude or even direction in humans [154,217]. Therefore, outcomes derived exclusively from animal studies, such as histological recovery patterns or molecular biomarkers (e.g., PGC-1α, sirtuin 1, or oxidative stress markers), should be interpreted cautiously when inferring potential clinical benefits. Future research should emphasize translational approaches, including well-controlled clinical studies, to validate these findings and clarify species-specific responses.

Recognizing these translational limitations helps to better define the next steps for future research. Building upon these considerations, substantial progress has been made in understanding the mechanisms of cisplatin-induced muscle atrophy and the protective effects of exercise, several gaps remain to be addressed [218,219]. Future research should focus on the following directions (Table 3):

Although patient heterogeneity necessitates personalized exercise prescriptions, the lack of standardized exercise protocols reflects the current gap between individualized practice and evidence-based framework development [227,228]. Bridging this gap through flexible standardized models should be a key goal for future research.

### 6.1. Standardization of Preclinical Models

Current evidence on cisplatin-induced muscle wasting is largely derived from heterogeneous animal models, with variations in drug dose, treatment duration, and muscle groups analyzed [221,225]. This lack of standardization complicates cross-study comparisons and limits translation to clinical settings [229]. Establishing standardized protocols for cisplatin administration, coupled with reproducible exercise interventions, will be essential to improve the reliability of preclinical findings [230].

### 6.2. Mechanistic Integration of Exercise Modalities

Most studies focus on either endurance or resistance training, whereas few directly compare or combine modalities in the context of chemotherapy-induced muscle atrophy [35,231]. Moreover, the underlying molecular crosstalk between endurance- and resistance-induced pathways remains poorly defined [10]. Future investigations should explore how combined training orchestrates proteostasis, mitochondrial function, and inflammation in an integrated manner, potentially revealing synergistic mechanisms beyond additive effects.

### 6.3. Translational Biomarkers and Myokine Profiling

Although several myokines such as irisin, IL-15, and FGF21 have been implicated in exercise-mediated protection, their roles as circulating biomarkers in chemotherapy patients remain to be validated [43,232]. Large-scale studies integrating omics approaches (proteomics [233], metabolomics [234], single-cell transcriptomics [235]) are needed to identify biomarker panels that reliably reflect muscle health and therapeutic responsiveness. This would also accelerate the development of pharmacological mimetics that reproduce the systemic benefits of exercise [236,237].

### 6.4. Clinical Implementation and Personalized Exercise Prescriptions

Despite encouraging preclinical evidence [189], clinical trials testing exercise interventions during cisplatin-based chemotherapy remain scarce [189,220]. Challenges include patient heterogeneity, treatment side effects, and adherence to exercise programs [220]. Future clinical studies should prioritize the development of personalized exercise prescriptions, accounting for cancer type, treatment regimen, baseline physical status, and genetic background. Integrating digital health tools such as wearables and tele-rehabilitation may enhance feasibility and patient compliance [222,223,224].

Moreover, recent studies highlight that skeletal muscle is not a uniform tissue but comprises distinct molecular subtypes exhibiting differential vulnerability to catabolic stress [87,238]. For example, Bhatt et al. identified molecular subtypes of human skeletal muscle in cancer cachexia, characterized by unique metabolic and inflammatory signatures that influence susceptibility to atrophy [87]. This molecular heterogeneity may also exist in chemotherapy-induced muscle wasting, potentially explaining interindividual variability in treatment response and exercise efficacy [29,35]. Incorporating this concept into future translational research could guide the development of more precise and subtype-specific exercise prescriptions.

### 6.5. Dietary Modulation in Chemotherapy-Induced Skeletal Muscle Atrophy

Recent findings emphasize that nutritional modulation plays a critical role in counteracting cisplatin-induced skeletal muscle wasting [239]. High-quality protein and branched-chain amino acid (BCAA) supplementation can enhance muscle protein synthesis through activation of the mTOR pathway and suppression of proteolysis [240]. Omega-3 polyunsaturated fatty acids and antioxidant vitamins (C and E) mitigate oxidative stress and inflammation, helping to preserve mitochondrial integrity and function [241]. Additionally, micronutrients such as selenium and zinc strengthen antioxidant defense systems [242,243], while polyphenolic compounds like resveratrol stimulate PGC-1α–mediated mitochondrial biogenesis and muscle regeneration [244]. Recent preclinical data also suggest that combining dietary support with exercise synergistically improves muscle recovery and metabolic adaptation in cisplatin-treated models [239]. Therefore, dietary intervention—particularly amino acid, antioxidant, and micronutrient supplementation—represents a promising adjunct strategy to attenuate chemotherapy-induced muscle atrophy [245].

### 6.6. Future Directions

In summary, future research should aim to (1) standardize preclinical models, (2) clarify the integrated mechanisms of different exercise modalities, (3) establish reliable biomarkers such as myokines, and (4) translate preclinical insights into personalized clinical interventions. Addressing these challenges will not only deepen mechanistic understanding but also accelerate the implementation of exercise-based strategies to preserve skeletal muscle health in patients undergoing cisplatin chemotherapy [43,226,246].

## 7. Conclusions

Cisplatin-induced skeletal muscle atrophy represents a major clinical challenge that compromises treatment tolerance, immune competence, and quality of life in cancer patients. Current evidence indicates that exercise is a uniquely effective intervention, capable of targeting multiple molecular pathways simultaneously, including proteostasis, mitochondrial function, oxidative stress, and inflammatory signaling. Both endurance and resistance training, alone or in combination, confer protective benefits, while exercise-induced myokines provide a molecular bridge linking local muscle adaptation to systemic protection. Despite these advances, significant gaps remain in standardizing preclinical models, identifying translational biomarkers, and implementing personalized exercise prescriptions in clinical practice. Addressing these challenges will be critical for transforming exercise from a supportive measure into an evidence-based therapeutic strategy. Ultimately, integrating exercise into cancer care has the potential not only to preserve skeletal muscle health but also to improve overall patient outcomes during cisplatin-based chemotherapy.

## Figures and Tables

**Table 1:** Comparative features of cisplatin-induced versus other types of skeletal muscle atrophy.

Feature/Mechanism	Cisplatin-Induced Atrophy	Disuse/Denervation Atrophy	Cancer Cachexia/Systemic Atrophy	References
Primary cause	Chemotherapy toxicity and systemic stress	Mechanical unloading or loss of neural input	Chronic inflammation and tumor–host interaction	[16,20]
Mitochondrial effect	Direct mtDNA damage and oxidative stress via cisplatin–DNA adducts	Reduced mitochondrial activity due to inactivity	Impaired mitochondrial biogenesis secondary to inflammation	[1,77,86]
Inflammatory cytokines	Marked systemic IL-6 and TNF-α elevation following chemotherapy	Mild local inflammation	Severe systemic inflammation (IL-1β, IL-6, TNF-α)	[16,18]
Myokine regulation	Decreased secretion of IL-15 and irisin; imbalance in myostatin signaling	Slightly reduced myokine production due to inactivity	Altered myokine profile driven by tumor–host crosstalk	[89,90]
PGC-1α and mitochondrial biogenesis	Markedly suppressed, leading to energy deficit and impaired regeneration	Reduced but recoverable with reloading	Suppressed by chronic inflammatory signaling	[1,86]
Dominant mechanism	Dual mechanism: direct cytotoxic injury and systemic metabolic dysregulation	Local disuse-induced proteolysis	Systemic inflammation–driven catabolism	[1,20]
Reversibility	Partially reversible with exercise or nutritional interventions	Fully reversible with reloading	Poorly reversible without treating the underlying disease	[75,88,91]

Note: IL, interleukin; TNF-α, tumor necrosis factor alpha; mtDNA, mitochondrial DNA; ROS, reactive oxygen species; PGC-1α, peroxisome proliferator-activated receptor gamma coactivator 1-alpha.

**Table 2:** Clinical and preclinical evidence of cisplatin-induced muscle atrophy.

Study Type	Subjects	Key Findings	References
Clinical	Head and Neck Cancer Patients	Cisplatin chemotherapy results in significant muscle mass loss and muscle atrophy symptoms.	[1]
Clinical	Lung Cancer Patients	Cisplatin treatment leads to substantial muscle mass and strength decline, impacting quality of life.	[6]
Clinical	Ovarian Cancer Patients	Cisplatin treatment decreases muscle index, resulting in impaired physical function.	[53]
Preclinical	Mouse Model	Cisplatin induces muscle mass reduction, with a significant decrease in muscle fiber cross-sectional area.	[7]
Preclinical	Mouse Model	Cisplatin causes a decrease in muscle protein synthesis, accompanied by activation of the protein degradation system.	[16]
Preclinical	Mouse Model	Cisplatin induces muscle atrophy with increased oxidative stress and inflammation.	[18]

**Table 3:** Challenges and future directions for clinical implementation of exercise interventions.

Challenges	Future Directions	References
Patient heterogeneity	Develop personalized exercise prescriptions tailored to cancer type, treatment regimen, baseline physical status, and genetic background.	[220,221,222]
Treatment side effects	Focus on understanding how different exercise modalities can reduce treatment-related muscle wasting and improve overall health.	[9,10,34]
Adherence to exercise programs	Integrate digital health tools, such as wearables and tele-rehabilitation, to enhance feasibility and patient compliance.	[220,223,224]
Lack of standardized exercise protocols	Standardize exercise protocols and establish reproducible interventions in preclinical and clinical research.	[220,225,226]
Limited clinical trials on exercise interventions	Conduct large-scale clinical trials to validate the efficacy of exercise interventions during cisplatin-based chemotherapy.	[189,219,220]

## Data Availability

Not applicable.
